# Emergence of *Vibrio cholerae* O1 Sequence Type 75 in Taiwan

**DOI:** 10.3201/eid2601.190934

**Published:** 2020-01

**Authors:** Yueh-Hua Tu, Bo-Han Chen, Yu-Ping Hong, Ying-Shu Liao, Yi-Syong Chen, Yen-Yi Liu, Ru-Hsiou Teng, You-Wun Wang, Chien-Shun Chiou

**Affiliations:** Centers for Disease Control, Taichung, Taiwan

**Keywords:** Bacteria, cholera, core genome MLST, cgMLST, molecular epidemiology, multilocus sequence typing, MLST, Taiwan, *Vibrio cholerae*, whole-genome sequencing, enteric infections

## Abstract

We investigated the epidemiology of cholera in Taiwan during 2002–2018. *Vibrio cholerae* sequence type (ST) 75 clone emerged in 2009 and has since become more prevalent than the ST69 clone from a previous pandemic. Closely related ST75 strains have emerged in 4 countries and may now be widespread in Asia.

Cholera, an acute diarrheal disease caused by the toxigenic *Vibrio cholerae* serogroup O1 and its derivative serogroup O139, remains a severe public health threat in some regions of the world (*1*). Seven cholera pandemics have occurred in the past 200 years; the most recent, caused primarily by a sequence type (ST) 69 *V. cholerae* clone, originated in Indonesia in 1961 and remains ongoing (*2*,*3*). In Taiwan, cholera appeared in 1962 and resulted in 383 cases and 24 deaths during a 3-month outbreak (*4*). Cholera has been rare in Taiwan since the 1962 outbreak; however, incidence has increased in recent years. The average number of cholera cases increased from 1.5 cases/year in 1991–2008 to 5.5 cases/year in 2009–2018 (*5*).

For this study, we investigated the epidemiology of cholera in Taiwan for 2002–2018 and the source of *V. cholerae* strains in those cases. During 2002–2018, Taiwan reported 63 total cholera cases, ranging from 0 to 10 cases per year (Table). Among the patients, 62 were from Taiwan and 1 from Japan; 35 (55.6%) were male. Three (4.8%) patients were in the <14 year age range, 37 (58.7%) in the 15–64 age range, and 23 (36.5%) in the >65 age range. Nearly all cases, 61, were sporadic; 2 were part of a family cluster. Seven patients had traveled within the incubation period (5 days) before onset of symptoms: 2 to Indonesia, 1 to Malaysia, 1 to Thailand, 2 to the Philippines, and 1 to Vietnam.

**Table:** Distribution of cholera and sequence types of *Vibrio cholerae* isolates, Taiwan, 1962 and 2002–2018

Category	1962	2002	2003	2004	2005	2006	2007	2008	2009	2010	2011	2012	2013	2014	2015	2016	2017	2018	Total
No. cases	383	2	1	1	2	1	0	1	3	5	3	5	7	4	10	9	2	7	63
No. isolates	4	2	1	1	2	1	0	1	3	4	3	3	6	3	8	9	2	7	56
Sequence type																			
69	4		1	1	2	1		1		1	1		2			3	1		14
75 and variants									3	3	2	3	4	3	8	6	1	7	40
723		2																	2

Using pulsed-field gel electrophoresis (PFGE) and whole-genome sequencing analysis, we characterized 60 *V. cholerae* isolates: 4 recovered from patients of the 1962 cholera outbreak and 56 recovered from patients during 2002–2018. Using PFGE, the 2 most prevalent isolates we identified were VcN09.014 (n = 25) and VcN09.012 (n = 7) (Appendix Figure 1). We compared those PFGE patterns with those in the *Vibrio cholerae* database maintained by the US Centers for Disease Control and Prevention in 2016 and found no match for VcN09.014, but we did find 11 isolates from Guam and the Philippines that matched with VcN09.012 (identification no. KZGN11.0102).

We identified 7 ST types for the 60 isolates: ST69 (18 isolates), ST723 (2 isolates), ST75 (35 isolates), ST725 (1 isolate), ST726 (2 isolates), ST727 (1 isolate), and ST728 (1 isolate). ST725, ST726, and ST728 are single-locus variants and ST727 a double-locus variant of ST75. ST75 and its derivatives first appeared in 2009 and subsequently become the most prevalent types (Table). Among the 7 patients who had traveled abroad, we found ST69 in those returning from Thailand, the Philippines, and Malaysia; ST75 in the person returning from Vietnam; and ST723 in those returning from Indonesia. We performed cluster analyses on whole-genome single nucleotide polymorphism profiles for the 60 isolates, which revealed 4 distinct clades (Appendix Table, Figure 2). The fact that a total of 5 isolates in clades 2 and 3 did not harbor the quinolone-resistant gene *qnrVC4* suggests that the resistance gene was introduced after the ST75 strains had emerged.

We compared core genome multilocus sequence typing profiles of the 60 isolates with 5,048 genomes in the National Center for Biotechnology Information databases as of January 19, 2019. We found that the 38 isolates in clade 2 were closely related to 10 strains from China (*6*), strain MS6 that was identified in Thailand in 2008 (*7*), and a UK strain recovered from a traveler who returned from Thailand in 2017 (*8*) (Appendix Figure 3). Strains from the 2 isolates in clade 3 were distantly removed from strains in clade 2 and those found near the US Gulf Coast (*9**,**10*) but more closely related to a strain recovered in 2018 and another strain from Russia. The ST75 strains from China were recovered from well water, carriers, and patients during 2005–2014 (*6*). One ST75 strain in clade 2 was obtained from a Taiwanese person who returned from Vietnam in 2015.

In summary, for most of recent history, cholera has been rare and primarily sporadic in Taiwan. However, the per-year rate of cholera cases has increased since 2009, concurrent with the emergence of strains of the ST75 clone. Over this time, ST75 strains have replaced ST69 as the most prevalent causative agent of cholera in Taiwan. Because closely related ST75 strains had been identified earlier in China and 2 other Southeast Asia countries, we believe our findings indicate that the ST75 clone is spreading more widely in Asia.

Appendix Additional information on emergence of *Vibrio cholerae* O1 sequence type 75 in Taiwan.

## References

[1] World Health Organization. Cholera Annual Report 2017. Wkly Epidemiol Rec. 2018;93:489–500.

[2] Mutreja A, Kim DW, Thomson NR, Connor TR, Lee JH, Kariuki S, et al. Evidence for several waves of global transmission in the seventh cholera pandemic. Nature. 2011;477:462–5. 10.1038/nature10392 21866102 PMC3736323

[3] Lippi D, Gotuzzo E, Caini S. Cholera. Microbiol Spectr. 2016;4. 10.1128/microbiolspec.PoH-0012-2015 27726771

[4] Yen CH. A recent study of cholera with reference to an outbreak in Taiwan in 1962. Bull World Health Organ. 1964;30:811–25. 10.1128/microbiolspec.PoH-0012-2015 14215187 PMC2555076

[5] Taiwan National Infectious Disease Statistics System [cited 2019 Feb 1]. https://nidss.cdc.gov.tw

[6] Luo Y, Octavia S, Jin D, Ye J, Miao Z, Jiang T, et al. US Gulf-like toxigenic O1 *Vibrio cholerae* causing sporadic cholera outbreaks in China. J Infect. 2016;72:564–72. 10.1016/j.jinf.2016.02.005 26920786

[7] Okada K, Na-Ubol M, Natakuathung W, Roobthaisong A, Maruyama F, Nakagawa I, et al. Comparative genomic characterization of a Thailand-Myanmar isolate, MS6, of *Vibrio cholerae* O1 El Tor, which is phylogenetically related to a “US Gulf Coast” clone. PLoS One. 2014;9:e98120. 10.1371/journal.pone.0098120 24887199 PMC4045137

[8] Greig DR, Schaefer U, Octavia S, Hunter E, Chattaway MA, Dallman TJ, et al. Evaluation of whole-genome sequencing for identification and typing of *Vibrio cholerae.* J Clin Microbiol. 2018;56:e00831–18. 10.1128/JCM.00831-18 30135231 PMC6204685

[9] Watve SS, Chande AT, Rishishwar L, Mariño-Ramírez L, Jordan IK, Hammer BK. Whole-genome sequences of 26 *Vibrio cholerae* isolates. Genome Announc. 2016;4:e01396–16. 10.1128/genomeA.01396-16 28007852 PMC5180380

[10] Kaper JB, Bradford HB, Roberts NC, Falkow S. Molecular epidemiology of *Vibrio cholerae* in the U.S. Gulf Coast. J Clin Microbiol. 1982;16:129–34. 7107852 10.1128/jcm.16.1.129-134.1982 PMC272308

