# Supplementary material for: Emergence of Vibrio cholerae O1 Sequence Type 75 in Taiwan
Source: Emerg Infect Dis. 2020 Jan;26(1):164–6. doi: 10.3201/eid2601.190934 (PMC6924918; doi:10.3201/eid2601.190934)
Supplement: Appendix — Additional information on emergence of Vibrio cholerae O1 sequence type 75 in Taiwan. [file 19-0934-Techapp-s1.pdf]

Article DOI: https://doi.org/10.3201/eid2601.190934

Emergence of *Vibrio cholerae* O1 Sequence Type 75 in Taiwan

Appendix

Experimental Methods

Cholera information and bacterial isolates

We obtained the statistical, demographic, and epidemiologic information on cholera cases in Taiwan from the databases of Taiwan National Infectious Disease Statistics System (https://nidss.cdc.gov.tw/en/) and the National Notifiable Diseases of Surveillance System (*1*) of the Taiwan Centers for Disease Control and *V. cholerae* isolates from the biobank section of Taiwan Centers for Disease Control. A total of 60 isolates were obtained for the study, among which 56 were recovered from cholera patients between 2002 and 2018 and 4 from patients of the cholera outbreak that occurred in 1962 (*2*).

Pulsed-field gel electrophoresis and analysis

We used the PulseNet standardized pulsed-field gel electrophoresis (PFGE) protocol (*3*) to characterize *V. cholerae* isolates, then analyzed PFGE patterns and performed clustering analysis of PFGE patterns using tools provided by BioNumerics 7.6.3 (Applied Maths; http://www.applied-maths.com).

Whole-genome sequencing and sequence analysis

We conducted whole-genome sequencing of *V. cholerae* isolates using Illumina MiSeq sequencing platform (Illumina Inc. USA) with MiSeq Reagent Kit v3 (2X 300 bp), assembled sequence reads using the SPAdes assembler version 3.12.0 (http://cab.spbu.ru/software/spades/), identified sequence types (STs) using the *Vibrio cholerae* core genome multilocus sequence typing MLST database (https://pubmlst.org/v*cholerae*) and antimicrobial resistance genes using the ResFinder tool of the Center for Genomic Epidemiology (http://www.genomicepidemiology.org/).

Construction of a whole genome single nucleotide polymorphism tree for *V. cholerae* strains from Taiwan

We used BioNumerics version 7.6.3 to construct a minimal spanning tree with whole genome single nucleotide polymorphism (wgSNP) profiles of *V. cholerae* strains. The sequences of raw reads were mapped to the reference genomic sequence of *V. cholerae* strain N16961 (GenBank accession no. GCA_000006745.1) and the mapped sequences of strains and the reference were aligned for SNP calling by using the option of strict SNP filtering (closed SNP set). By using this SNP calling criteria, SNPs are called by removing positions with at least one ambiguous base (non-ATGC base), one unreliable base (N), one gap and non-informative SNPs. Each retained SNP position has minimum 5x coverage, at least covered once in both forward and reverse direction. The minimum distance between retained SNP positions is 12 bp. A dendrogram was constructed with the whole genome SNP profiles using the categorical (SNPs) option for similarity coefficient and minimum spanning tree algorithm for cluster analysis.

Construction of a core genome multilocus sequence typing tree for *V. cholerae* strains from Taiwan and NCBI database

We downloaded genomic sequences from the SRA and Assembly databases of the National Center for Biotechnology Information (NCBI), assembled raw reads using the SPAdes assembler version 3.12.0, and generated core genome multilocus sequence typing (cgMLST) profiles (based on 2,951 core genes) using the in-house developed cgMLST Profiling tool in the cgMLST@Taiwan Web service platform (https://rdvd.cdc.gov.tw/cgMLST) (unpublished). We compared the cgMLST profiles of 60 isolates from Taiwan with those (5,048) from the NCBI database and selected the most match profiles with the Taiwanese isolates to construct a cgMLST genetic relatedness tree using the unweighted pair-group method with arithmetic means algorithm.

References

1. Jian SW, Chen CM, Lee CY, Liu DP. Real-time surveillance of infectious diseases: Taiwan's experience. Health Secur. 2017;15:144–53.

2. Yen CH. A recent study of cholera with reference to an outbreak in Taiwan in 1962. Bull World Health Organ. 1964;30:811–25.

3. Cooper KL, Luey CK, Bird M, Terajima J, Nair GB, Kam KM, et al. Development and validation of a PulseNet standardized pulsed-field gel electrophoresis protocol for subtyping of *Vibrio cholerae*. Foodborne Pathog Dis. 2006;3:51–8.

Appendix Table. Details of *Vibrio cholerae* strains from cholera cases in Taiwan, 2002–2018

Clade	Sequence type	Variants	No. of isolates	Resistance genes	Country of origin
1	ST69		18	*catB9*§	Taiwan, Thailand, Philippines, Malaysia
2	ST75	ST725*, ST726*, ST728*, ST727†	38	*qnrVC4*¶ (in 35 of 38 isolates)	Taiwan, Vietnam
3	ST75		2	None	Taiwan
4	ST723		2‡	None	Indonesia

*Single-locus variant
†Double-locus variant
‡Nontoxigenic
§Resistant to chloramphenicol
¶Resistant to quinolone

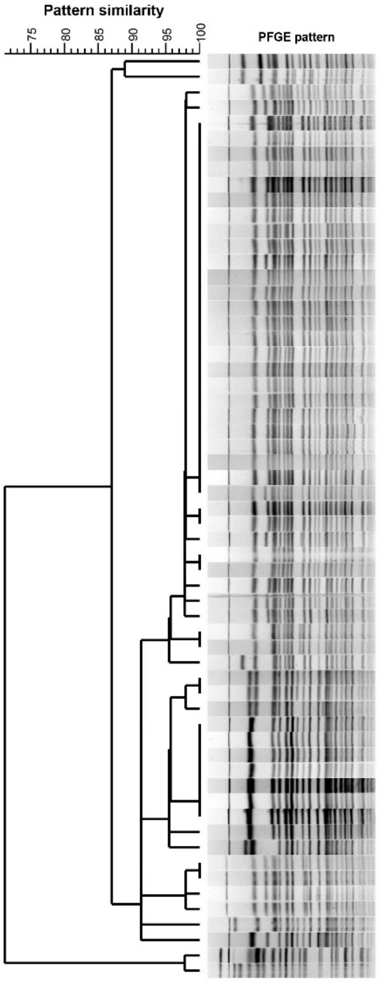

StrainID	BioSample	Year	Origin	Serovar	ctx	PFGE	ST	Clade	Resistance gene
R16.3429	SAMN09982357	2010	Taiwan	O1_Ogawa	Yes	VcN09.028	75	III	
R16.3447	SAMN09982367	2013	Taiwan	O1_Ogawa	Yes	VcN09.029	75	III	
R16.3223	SAMN09982349	2016	Taiwan	O1_Ogawa	Yes	VcN09.016	75	II	*qnrVC4*
R16.4299	SAMN09982375	2016	Taiwan	O1_Ogawa	Yes	VcN09.040	75	II	*qnrVC4*
R16.3057	SAMN09982348	2016	Taiwan	O1_Ogawa	Yes	VcN09.014	75	II	
R16.3446	SAMN09982366	2013	Taiwan	O1_Ogawa	Yes	VcN09.014	75	II	*qnrVC4*
R18.2632	SAMN11053874	2018	Taiwan	O1_Ogawa	Yes	VcN09.014	75	II	*qnrVC4*
R16.3423	SAMN09982352	2009	Taiwan	O1_Ogawa	Yes	VcN09.014	75	II	*qnrVC4*
R16.3022	SAMN09982344	2016	Taiwan	O1_Ogawa	Yes	VcN09.014	75	II	*qnrVC4*
R18.2633	SAMN11053875	2018	Taiwan	O1_Ogawa	Yes	VcN09.014	75	II	*qnrVC4*
R17.6060	SAMN09982384	2017	Taiwan	O1_Ogawa	Yes	VcN09.014	75	II	*qnrVC4*
R16.3439	SAMN09982360	2011	Taiwan	O1_Ogawa	Yes	VcN09.014	727	II	*qnrVC4*
R16.3427	SAMN09982355	2010	Taiwan	O1_Ogawa	Yes	VcN09.014	75	II	*qnrVC4*
R16.3449	SAMN09982369	2014	Taiwan	O1_Ogawa	Yes	VcN09.014	726	II	*qnrVC4*
C12.0862	SAMN09982338	2012	Taiwan	O1_Ogawa	Yes	VcN09.014	75	II	*qnrVC4*
R18.1640	SAMN09982386	2018	Taiwan	O1_Ogawa	Yes	VcN09.014	75	II	*qnrVC4*
R18.2631	SAMN11053873	2018	Taiwan	O1_Ogawa	Yes	VcN09.014	75	II	*qnrVC4*
C15.0632	SAMN11053863	2015	Taiwan	O1_Ogawa	Yes	VcN09.014	75	II	*qnrVC4*
R16.3451	SAMN09982371	2015	Taiwan	O1_Ogawa	Yes	VcN09.014	75	II	*qnrVC4*
R16.3448	SAMN09982368	2014	Taiwan	O1_Ogawa	Yes	VcN09.014	75	II	*qnrVC4*
R16.3443	SAMN11053865	2013	Taiwan	O1_Ogawa	Yes	VcN09.014	728	II	*qnrVC4*
R16.3425	SAMN09982354	2009	Taiwan	O1_Inaba	Yes	VcN09.014	75	II	*qnrVC4*
R16.3438	SAMN09982359	2011	Taiwan	O1_Ogawa	Yes	VcN09.014	75	II	*qnrVC4*
R16.3452	SAMN09982372	2015	Taiwan	O1_Ogawa	Yes	VcN09.014	75	II	*qnrVC4*
R16.3454	SAMN09982374	2015	Taiwan	O1_Ogawa	Yes	VcN09.014	75	II	*qnrVC4*
R16.3453	SAMN09982373	2015	Vietnam	O1_Ogawa	Yes	VcN09.014	75	II	*qnrVC4*
C12.0861	SAMN09982337	2012	Taiwan	O1_Ogawa	Yes	VcN09.014	75	II	*qnrVC4*
R16.5188	SAMN09982383	2015	Taiwan	O1_Ogawa	Yes	VcN09.014	75	II	*qnrVC4*
R18.2413	SAMN11053870	2018	Taiwan	O1_Ogawa	Yes	VcN09.014	75	II	*qnrVC4*
R16.3424	SAMN09982353	2009	Taiwan	O1_Ogawa	Yes	VcN09.020	725	II	*qnrVC4*
R16.5186	SAMN09982381	2015	Taiwan	O1_Ogawa	Yes	VcN09.020	75	II	*qnrVC4*
R16.3450	SAMN09982370	2014	Taiwan	O1_Ogawa	Yes	VcN09.042	75	II	*qnrVC4*
R16.4831	SAMN11053866	2016	Taiwan	O1_Ogawa	Yes	VcN09.021	75	II	*qnrVC4*
R18.2414	SAMN11053871	2018	Taiwan	O1_Ogawa	Yes	VcN09.021	75	II	*qnrVC4*
R16.5187	SAMN09982382	2015	Taiwan	O1_Ogawa	Yes	VcN09.015	75	II	*qnrVC4*
R16.3440	SAMN09982361	2012	Taiwan	O1_Ogawa	Yes	VcN09.044	75	II	*qnrVC4*
R16.3428	SAMN09982356	2010	Taiwan	O1_Ogawa	Yes	VcN09.019	726	II	*qnrVC4*
R16.3023	SAMN09982345	2016	Taiwan	O1_Ogawa	Yes	VcN09.013	75	II	
R18.2415	SAMN11053872	2018	Taiwan	O1_Ogawa	Yes	VcN09.013	75	II	
R16.3445	SAMN09982365	2013	Taiwan	O1_Ogawa	Yes	VcN09.043	75	II	*qnrVC4*
R16.3416	SAMN11053864	2005	Taiwan	O1_Ogawa	Yes	VcN09.023	69	I	*catB9*
R16.5185	SAMN11053869	2005	Taiwan	O1_Ogawa	Yes	VcN09.023	69	I	*catB9*
R18.2636	SAMN11053876	2003	Thailand	O1_Inaba	Yes	VcN09.024	69	I	*catB9*
R16.3442	SAMN09982363	2013	Taiwan	O1_Ogawa	Yes	VcN09.012	69	I	*catB9*
R16.2076	SAMN09982342	2016	Taiwan	O1_Ogawa	Yes	VcN09.012	69	I	*catB9*
R19.0132	SAMN11174976	2004	Philippines	O1_Ogawa	Yes	VcN09.012	69	I	*catB9*
R17.6061	SAMN09982385	2017	Philippines	O1_Ogawa	Yes	VcN09.012	69	I	*catB9*
R16.3441	SAMN09982362	2013	Taiwan	O1_Ogawa	Yes	VcN09.012	69	I	*catB9*
R16.2277	SAMN09982343	2016	Taiwan	O1_Ogawa	Yes	VcN09.012	69	I	*catB9*
R16.2075	SAMN09982341	2016	Taiwan	O1_Ogawa	Yes	VcN09.012	69	I	*catB9*
R16.3421	SAMN09982351	2008	Taiwan	O1_Ogawa	Yes	VcN09.025	69	I	*catB9*
R18.2638	SAMN11053878	2010	Taiwan	O139	Yes	VcN09.059	69	I	*catB9, aac(3)-IId, aph(3')-Ia, aph(6)-Id,* bla_{TEM-1B}*, sul2, tet(D), dfrA12*
R16.5177	SAMN09982380	1962	Taiwan	O1_Ogawa	Yes	VcN09.041	69	I	*catB9*
R16.5167	SAMN09982377	1962	Taiwan	O1_Ogawa	Yes	VcN09.041	69	I	*catB9*
R16.5176	SAMN09982379	1962	Taiwan	O1_Ogawa	Yes	VcN09.052	69	I	*catB9*
R16.5166	SAMN09982376	1962	Taiwan	O1_Ogawa	Yes	VcN09.051	69	I	*catB9*
R16.3431	SAMN09982358	2011	Malaysia	O1_Ogawa	Yes	VcN09.026	69	I	*catB9*
R18.2637	SAMN11053877	2006	Taiwan	O139	Yes	VcN09.027	69	I	*catB9, aac(3)-IId, aph(3')-Ia, aph(6)-Id,* bla_{TEM-1B}*, floR, sul2, tet(D), dfrA12*
R16.5170	SAMN11053867	2002	Indonesia	O1_Ogawa	No	VcN09.061	723	VI	*aadA2b, aph(3')-Ia,* bla_{CARB-2}*, mph(A), floR, sul1, tet(G)*
R16.5171	SAMN11053868	2002	Indonesia	O1_Ogawa	No	VcN09.062	723	VI	*aadA2b, aph(3')-Ia,* bla_{CARB-2}*, mph(A), floR, sul1, tet(G)*

Appendix Figure 1. Genetic relationships among 60 Vibrio cholerae isolates, with corresponding information. The dendrogram was constructed using PFGE patterns and the single linkage algorithm provided in BioNumerics software version 7.6.3, with settings of 1.5% optimization and 0.95% tolerance.

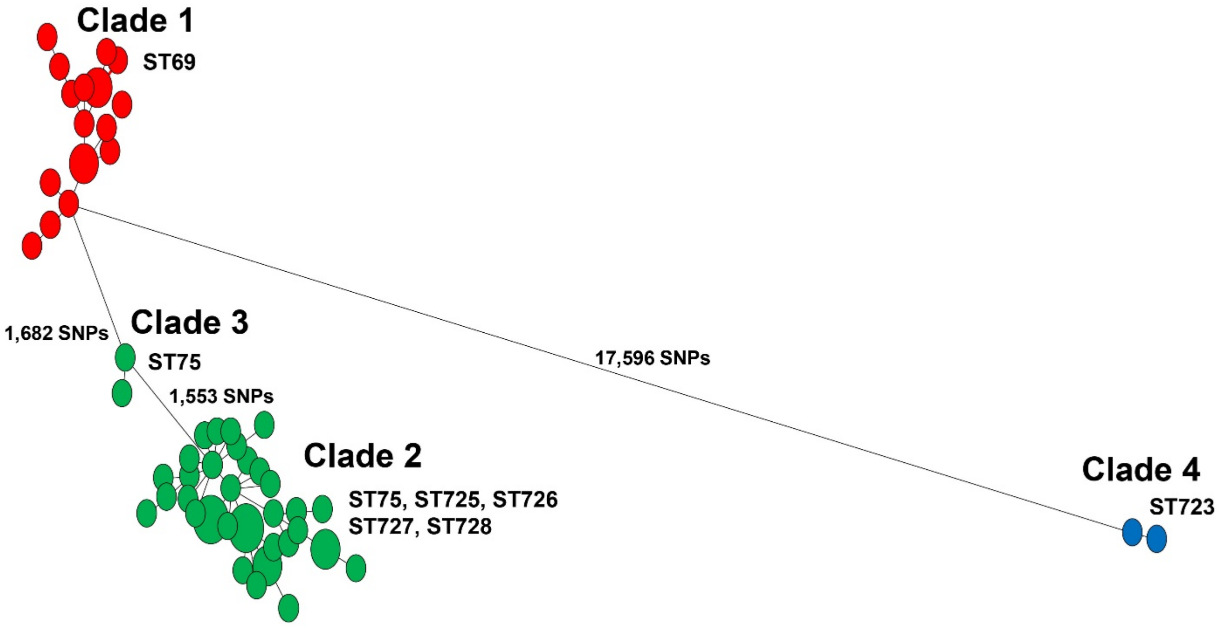

Appendix Figure 2. A minimum spanning tree for 60 Vibrio cholerae isolates from Taiwan collected in 1962 and in 2002–2018. The tree was constructed with whole genome single-nucleotide polymorphism profiles comprising 20,639 SNPs.

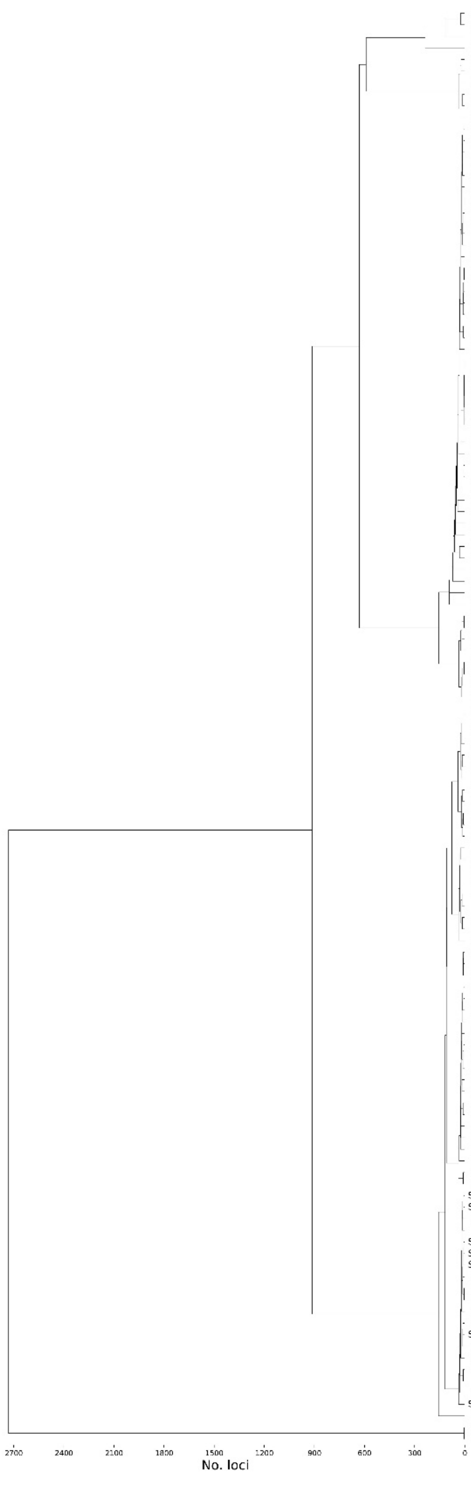

BioSample	SourceSeq.	StrainID/Alias	IsolatYear	SourceCountry	Serogroup_type	ST	No. voidloci
SAMN09982367	SRR7791616	R16.3447	2013	Taiwan	O1_Ogawa	75	5
SAMN09982357	SRR7791601	R16.3429	2010	Taiwan	O1_Ogawa	75	6
SAMN10390798	SRR8169152		2018			75	7
SAMN03234995	GCA_000818865.1	I-1471	2011	Russia	O1_Ogawa	75	17
SAMN05590418	SRR4039808	2497-86	1987	USA	O1	75	0
SAMN05590417	SRR4039807	1496-86	1986	USA	O1	75	0
SAMN02943003	GCA_001683415.1	2740-80	1980	USA	O1_Inaba	75	0
SAMN05590413	SRR4039827	2512-86	1986	USA	O1	75	2
SAMN05590410	SRR4039825	2559-78	1978	USA	O1	75	0
SAMN04256436	GCA_001887475.1	E506	1973	USA	O1	75	0
SAMN09982337	SRR7791632	C12.0861	2012	Taiwan	O1_Ogawa	75	4
SAMN09982338	SRR7791629	C12.0862	2012	Taiwan	O1_Ogawa	75	4
SAMN09982365	SRR7791593	R16.3445	2013	Taiwan	O1_Ogawa	75	4
SAMN09982352	SRR7791607	R16.3423	2009	Taiwan	O1_Ogawa	75	5
SAMN04196979	SRR2738136	ZJ1336	2005	China	O1_Inaba	75	4
SAMN09982354	SRR7791621	R16.3425	2009	Taiwan	O1_Inaba	75	4
SAMN09982382	SRR7791642	R16.5187	2015	Taiwan	O1_Ogawa	75	4
SAMN09982381	SRR7791639	R16.5186	2015	Taiwan	O1_Ogawa	75	4
SAMN09982383	SRR7791641	R16.5188	2015	Taiwan	O1_Ogawa	75	4
SAMN09982359	SRR7791599	R16.3438	2011	Taiwan	O1_Ogawa	75	4
SAMN09982355	SRR7791622	R16.3427	2010	Taiwan	O1_Ogawa	75	4
SAMN11053865	SRR8668522	R16.3443	2013	Taiwan	O1_Ogawa	728	13
SAMN04196974	SRR2738131	ZJ0926	2009	China	O1_Ogawa	75	6
SAMN04196973	SRR2738130	ZJ0921	2009	China	O1_Ogawa	75	6
SAMN04196977	SRR2738134	ZJ0946	2009	China	O1_Inaba	75	6
SAMN04196975	SRR2738132	ZJ0937	2009	China	O1_Ogawa	75	7
SAMN04196976	SRR2738133	ZJ0938	2009	China	O1_Ogawa	75	8
SAMN11053866	SRR8668521	R16.4831	2016	Taiwan	O1_Ogawa	75	4
SAMN11053871	SRR8668514	R18.2414	2018	Taiwan	O1_Ogawa	75	0
SAMN09982361	SRR7791597	R16.3440	2012	Taiwan	O1_Ogawa	75	4
SAMN09982373	SRR7791605	R16.3453	2015	Taiwan/Vietnam	O1_Ogawa	75	4
SAMN09982374	SRR7791592	R16.3454	2015	Taiwan	O1_Ogawa	75	4
SAMN09982371	SRR7791620	R16.3451	2015	Taiwan	O1_Ogawa	75	4
SAMN09982368	SRR7791617	R16.3448	2014	Taiwan	O1_Ogawa	75	4
SAMN09982372	SRR7791625	R16.3452	2015	Taiwan	O1_Ogawa	75	4
SAMN11053873	SRR8668528	R18.2631	2018	Taiwan	O1_Ogawa	75	0
SAMN09982344	SRR7791623	R16.3022	2016	Taiwan	O1_Ogawa	75	4
SAMN11053870	SRR8668517	R18.2413	2018	Taiwan	O1_Ogawa	75	0
SAMN09982366	SRR7791615	R16.3446	2013	Taiwan	O1_Ogawa	75	4
SAMN04196972	SRR2738129	ZJ0917	2009	China	O1_Ogawa	75	5
SAMN04196971	SRR2738125	ZJ0911	2009	China	O1_Ogawa	75	7
SAMN11053872	SRR8668513	R18.2415	2018	Taiwan	O1_Ogawa	75	0
SAMN04196978	SRR2738135	ZJ10014	2010	China	O1_Ogawa	75	7
SAMD00061025	GCA_000829215.1	MS6	2008	Thailand	O1_Ogawa	75	4
SAMN08979236	SRR7062631		2017	Thailand	O1	75	6
SAMN04196970	SRR2738124	ZJ07019	2007	China	O1_Ogawa	75	4
SAMN09982369	SRR7791618	R16.3449	2014	Taiwan	O1_Ogawa	726	4
SAMN09982356	SRR7791602	R16.3428	2010	Taiwan	O1_Ogawa	726	4
SAMN11053863	SRR8668520	C15.0632	2015	Taiwan	O1_Ogawa	75	0
SAMN09982353	SRR7791608	R16.3424	2009	Taiwan	O1_Ogawa	725	5
SAMN09982360	SRR7791598	R16.3439	2011	Taiwan	O1_Ogawa	727	8
SAMN09982345	SRR7791624	R16.3023	2016	Taiwan	O1_Ogawa	75	5
SAMN11053875	SRR8668526	R18.2633	2018	Taiwan	O1_Ogawa	75	0
SAMN09982386	SRR7791606	R18.1640	2018	Taiwan	O1_Ogawa	75	5
SAMN09982384	SRR7791634	R17.6060	2017	Taiwan	O1_Ogawa	75	6
SAMN11053874	SRR8668527	R18.2632	2018	Taiwan	O1_Ogawa	75	0
SAMN09982370	SRR7791619	R16.3450	2014	Taiwan	O1_Ogawa	75	5
SAMN09982349	SRR7791612	R16.3223	2016	Taiwan	O1_Ogawa	75	5
SAMN09982375	SRR7791604	R16.4299	2016	Taiwan	O1_Ogawa	75	5
SAMN09982348	SRR7791611	R16.3057	2016	Taiwan	O1_Ogawa	75	6
SAMN09982343	SRR7791626	R16.2277	2016	Taiwan	O1_Ogawa	69	0
SAMN09982342	SRR7791603	R16.2076	2016	Taiwan	O1_Ogawa	69	0
SAMN09982341	SRR7791628	R16.2075	2016	Taiwan	O1_Ogawa	69	0
SAMN09982385	SRR7791633	R17.6061	2017	Taiwan/Philippines	O1_Ogawa	69	2
SAMN02352646	GCA_001027505.1	PhVC-311	2011	Philippines		69	2
SAMN02352647	GCA_001027485.1	PhVC-326	2011	Philippines		69	2
SAMN02352648	GCA_001027495.1	PhVE-5	2011	Philippines		69	1
SAMN11174976	SRR8755238	R19.0132	2004	Taiwan/Philippines	O1_Ogawa	69	0
SAMN09982351	SRR7791610	R16.3421	2008	Taiwan	O1_Ogawa	69	0
SAMN09982362	SRR7791596	R16.3441	2013	Taiwan	O1_Ogawa	69	0
SAMN09982363	SRR7791595	R16.3442	2013	Taiwan	O1_Ogawa	69	0
SAMN08979114	SRR7062500		2015	Asian continent	O1	69	0
SAMEA889303	GCA_001259055.1	4113	2003	Vietnam	O1_Inaba	69	0
SAMEA2693539	ERR576951	V011506	2001	China	O1_Inaba	69	1
SAMN11053864	SRR8668519	R16.3416	2005	Taiwan	O1_Ogawa	69	1
SAMN11053869	SRR8668518	R16.5185	2005	Taiwan	O1_Ogawa	69	1
SAMN11053876	SRR8668525	R18.2636	2003	Taiwan/Thailand	O1_Inaba	69	0
SAMEA889305	GCA_001252075.1	4121	2004	Vietnam	O1_Ogawa	69	0
SAMEA4057557	ERR1485209	22116	2004	Thailand	O1_Inaba	69	0
SAMEA4057556	ERR1485207	22115	2004	Thailand	O1_Inaba	69	0
SAMEA2698348	ERR579085	147-CN	2002	China	O1_Inaba	69	4
SAMN00138732	GCA_000166475.2	2010EL-1798	2010	Haiti	O1_Ogawa	69	0
SAMN00765529	GCA_000166455.2	2010EL-1786	2010	Haiti	O1_Ogawa	69	0
SAMN00138733	GCA_000166495.2	2010EL-1792	2010	Haiti	O1_Ogawa	69	1
SAMN04286040	SRR3020470	ICDC-VC1785	2003	China		69	2
SAMN04285993	SRR3020423	ICDC-VC2370	2007	China		69	1
SAMN04286008	SRR3020438	ICDC-VC1662	2006	China		69	1
SAMN04286000	SRR3020430	ICDC-VC2071	2005	China		69	3
SAMN04286007	SRR3020437	ICDC-VC1999	2001	China		69	1
SAMN04286015	SRR3020445	ICDC-VC1825	2006	China		69	1
SAMN04285988	SRR3020418	ICDC-VC1449	2006	China		69	2
SAMN04285986	SRR3020416	ICDC-VC2035	2006	China		69	2
SAMN04286016	SRR3020446	ICDC-VC2090	2007	China		69	2
SAMN04285985	SRR3020415	ICDC-VC4239	2007	China		69	3
SAMN04286005	SRR3020435	ICDC-VC4268	2008	China		69	1
SAMN04285984	SRR3020414	ICDC-VC4273	2008	China		69	3
SAMN04286010	SRR3020440	ICDC-VC381	2001	China		69	6
SAMN04286044	SRR3020474	ICDC-VC1275	2000	China		69	5
SAMN04286022	SRR3020452	ICDC-VC1464	2006	China		69	2
SAMN04286034	SRR3020464	ICDC-VC909	2002	China		69	6
SAMN11053877	SRR8668524	R18.2637	2006	Taiwan	O139	69	0
SAMN11053878	SRR8668523	R18.2638	2010	Taiwan	O139	69	0
SAMEA103907199	ERR1879629	CNRVC960976	1962	Papua New Guinea		69	0
SAMEA103905533	ERR1879565	CNRVC930061	1961	China		69	0
SAMN09982379	SRR7791637	R16.5176	1962	Taiwan	O1_Ogawa	69	1
SAMEA889329	GCA_001252495.1	A18	1977	India	O1_Inaba	69	0
SAMEA103907200	ERR1879630	CNRVC961005	1964	Pakistan		69	0
SAMEA103906388	ERR1879604	CNRVC960800	1965	Iran		69	0
SAMEA103906384	ERR1879601	CNRVC960768	1966	Iraq		69	2
SAMN08721838	GCA_003013485.1	2044	1966	Iraq		69	0
SAMN09982376	SRR7791636	R16.5166	1962	Taiwan	O1_Ogawa	69	0
SAMN09982377	SRR7791635	R16.5167	1962	Taiwan	O1_Ogawa	69	0
SAMN09982380	SRR7791640	R16.5177	1962	Taiwan	O1_Ogawa	69	0
SAMN04256434	GCA_001887435.1	CRC711	1964	India		69	0
SAMEA103905530	ERR1879562	CNRVC930036	1962	India		69	0
SAMN04256433	GCA_001887455.1	CRC1106	1962	India		69	0
SAMN04256439	GCA_001887495.1	E1162	1962	China		69	0
SAMN04256432	GCA_001887395.1	C5	1957	Indonesia		69	0
SAMEA889336	GCA_001255575.1	A6	1957	Indonesia	O1_Inaba	69	0
SAMN04256431	GCA_001887655.1	E9120	1961	Indonesia		69	0
SAMEA103905938	ERR1879583	CNRVC950172	1969	Vietnam		69	0
SAMN09982358	SRR7791600	R16.3431	2011	Taiwan/Malaysia	O1_Ogawa	69	29
SAMN11053867	SRR8668516	R16.5170	2002	Taiwan/Indonesia	O1_Ogawa	723	43
SAMN11053868	SRR8668515	R16.5171	2002	Taiwan/Indonesia	O1_Ogawa	723	42

Appendix Figure 3. A genetic dendrogram for 60 Vibrio cholerae isolates collected in Taiwan and their most closely related strains in the NCBI database. Three strains from the 2010 Haiti cholera outbreak were included for comparison. The tree was constructed with cgMLST profiles using the unweighted pair-group method with arithmetic means algorithm.
